# Clinical Memoranda from the Surgical Clinic at the Sisters of Charity Hospital

**Published:** 1894-05

**Authors:** Herman Mynter

**Affiliations:** Professor of Surgery, Niagara University, and Surgeon to the Sisters’ Hospital


### Clinical Report.

### CLINICAL MEMORANDA FROM THE SURGICAL CLINIC AT THE SISTERS OF CHARITY HOSPITAL.

By HERMAN MYNTER, M. D.,

Professor of Surgery, Niagara University, and Surgeon to the Sisters' Hospital.

### TUBERCULOUS EPIDIDYMITIS.

In a paper on Tuberculous Epididymitis, published in *Annals of Surgery*, in April, 1893, I advocated the early and complete extirpation of the tuberculous epididymis, from the following reasons: A patient with double tuberculous epididymitis is sterile and his semen does not contain spermatozoa. The testes must atrophy unless the worse thing happen that they become secondarily tuberculous. The danger of extension of the tuberculous process to the prostate, bladder and kidney is great, and the disease is then fatal. By extirpating the diseased epididymis we do not make the patient any more sterile than he was before, and by retaining the testes sufficient nervous stimulus is preserved to produce erection and make sexual intercourse possible ; yes, even an ejaculation, probably from vesiculæ seminales, may occur. This little operation is devoid of danger, and a sure and radical cure may be obtained in a couple of weeks. The cases I reported in 1893 are still in excellent health, and I wish here to report two other cases in support of this operation and earnestly to advocate its use:

Case XV.—Mr. W. C. F., twenty-three years of age, entered the Sisters' hospital, on April 17, 1893, with the following history: He was kicked on the left testis five years previously. The testis swelled, but after the swelling was reduced a lump was left around the left testis, which has since been the source of a great deal of pain and discomfort. He has never had gonorrhea or syphilis ; no tuberculosis in the family. On examination, the left epididymis was found nodulated, as large as a dove's egg ; the testis felt normal, with normal testicular sense, the vas deferens thickened and painful for several inches ; the vesiculæ seminales normal. Under cocaine anesthesia the left epididymis and four inches of the diseased vas deferens were removed. The wound healed by first intention and he left the hospital, recovered, on April 23d, and has been well since. The microscopical examination of the specimen showed tuberculous bacilli.

Case XVI.—Mr. D. M., aged thirty, entered the hospital on March 8, 1894. In August, 1893, he noticed that the right testicle was tender and painful, and a small hard lump appeared at its lower end. This lump increased in size for about seven weeks, when it broke, leaving a sinus, through which yellowish matter was discharged. The lump became thereafter smaller and harder. About the same time, a small, hard, painful swelling appeared at the lower end of the left testicle. This swelling remained stationary until about January 10, 1894, when it increased greatly in size and became intensely painful. At last it broke, leaving a sinus behind, through which a cheesy material may be pressed out. His general health is good ; no swelling of the vesiculæ seminales. Several members of the family had died of tuberculosis. As he himself had been unable to work since August last, he readily submitted to operation when its nature had been explained to him.

At the examination, both epididymes were found hard, painful and enlarged to about twice the size of the testes. Either testis was easily outlined and apparently healthy. Under narcosis, both epididymes, with several inches of the vas deferens, were removed. The epididymes were both found in a state of tuberculous degeneration with cheesy deposits and circumscribed abscesses, extending on the left side slightly into the hilus of the testes. This was carefully removed by sharp spoon, the wounds sutured and antiseptic bandages applied. He left the hospital on March 20th with the wounds healed by first intention. He called at my office on April 6th, stated that he felt well, had no pain whatever, could have strong erections, and had gone to work as brakeman on the railroad again. The testes felt normal, with testicular sense; over the left hilus still a little hardness.

---

The Pennsylvania Railroad Company has sent out a special train with two physicians, who are to go over the whole line to Chicago, and vaccinate at each station all the switchmen, sectionmen, gatekeepers and other employes.—*Chicago Medical Record.*

